# Polymyxins: To Combine or Not to Combine?

**DOI:** 10.3390/antibiotics8020038

**Published:** 2019-04-10

**Authors:** Federico Perez, Nadim G. El Chakhtoura, Mohamad Yasmin, Robert A. Bonomo

**Affiliations:** 1 Medicine Service, Louis Stokes Cleveland Veterans Affairs Medical Center, Cleveland, OH 44106, USA; nge11@case.edu (N.G.E.C.); mohamad.yasmin@uhhospitals.org (M.Y.); Robert.Bonomo@va.gov (R.A.B.); 2 Research Service, Louis Stokes Cleveland Veterans Affairs Medical Center, Cleveland, OH 44106, USA; 3 Geriatrics Research, Education and Clinical Center, Louis Stokes Cleveland Veterans Affairs Medical Center, Cleveland, OH 44106, USA; 4 Department of Medicine, University Hospitals Cleveland Medical Center, Cleveland, OH 44106, USA; 5 Case VA Center for Antimicrobial Resistance and Epidemiology, Cleveland, OH 44106, USA; 6 Department of Biochemistry, Case Western Reserve University School of Medicine, Cleveland, OH 44106, USA; 7 Department of Molecular Biology and Microbiology, Case Western Reserve University School of Medicine, Cleveland, OH 44106 USA; 8 Department of Pharmacology, Case Western Reserve University School of Medicine, Cleveland, OH 44106, USA

**Keywords:** polymyxin B, colistin, antibiotic-combinations, CRE

## Abstract

Polymyxins have been a mainstay for the treatment of extensively drug resistant (XDR) Gram-negative bacteria for the past two decades. Many questions regarding the clinical use of polymyxins have been answered, but whether the administration of polymyxins in combination with other antibiotics leads to better outcomes remains unknown. This review discusses the limitations of observational studies that suggest a benefit of combinations of colistin and carbapenems to treat infections caused by carbapenem-resistant Enterobacteriaceae (CRE), especially *Klebsiella pneumoniae* carbapenemase (KPC)-producing *K. pneumoniae*, and summarizes the results of randomized controlled trials in which treatment with colistin in combination with meropenem or rifampin does not lead to better clinical outcomes than colisitn monotherapy in infections caused by carbapenem-resistant *Acinetobacter baumannii* (CRAB). Although the introduction of new antibiotics makes it possible to treat certain strains of CRE and carbapenem-resistant *P. aeruginosa* (CRPA) with polymyxin-sparing regimens, the use of polymyxins is, for now, still necessary in CRAB and in CRE and CRPA harboring metallo-beta-lactamases. Therefore, strategies must be developed to optimize polymyxin-based treatments, informed by in vitro hollow fiber models, careful clinical observations, and high-quality evidence from appropriately designed trials.

## 1. Introduction

Polymyxins are cationic antimicrobial polypeptides that bind to negatively charged phosphate moieties in the lipid A fraction of lipopolysaccharide (LPS) present in the outer membrane of Gram-negative bacteria. As a result, polymyxins act by disrupting the bacterial cell membrane; this process results in the loss of intracellular products, therefore leading to bactericidal activity. The two examples of polymyxins used in the clinic, polymyxin B and polymyxin E (i.e., colistin), were introduced in the 1950s without the scrutiny of regulatory agencies. The toxicity profile and the subsequent development of aminoglycosides and cephalosporins relegated polymyxins to a secondary role. Nevertheless, polymyxins were “resurrected” at the end of the 20th century to address the therapeutic challenge posed by emergent extensively drug resistant (XDR) Gram-negative bacteria, especially carbapenem-resistant *Pseudomonas aeruginosa* (CRPA), carbapenem-resistant *Enterobacteriaceae* (CRE), and carbapenem-resistant *Acinetobacter baumannii* (CRAB) [1].

## 2. Polymyxins: Some Questions Answered and Emerging of Resistance

In the past 20 years, significant progress has been made in understanding the clinical pharmacology of polymyxins [2]. A deeper appreciation of the relative toxicity of polymyxin B and colistin, the variability of colistin preparations and their pharmacokinetics in critically ill patients, and dosing strategies that result in efficacious concentrations at the site of infection, have been at the forefront of the contemporary research agenda [3,4]. Consequently, there is consensus around the notion that polymyxin B may have pharmacologic characteristics that render it superior to colistin, and recognition of the role of polymyxin administration into the site of infection (e.g., intrathecal/intraventricular colistin for meningitis and ventriculitis) [5,6]. Similarly, progress in refining susceptibility testing methods and developing breakpoints of resistance for polymyxins has been made [7]. 

Various strategies to evade the bactericidal activity of polymyxins are found in bacteria. Adaptive mechanisms of polymyxin resistance in bacteria are governed by two-component systems, such as phoP/phoQ and pmrA/pmrB, which modify the charge of the bacterial membrane in response to low-magnesium concentrations and other environmental stimuli, including exposure to polymyxins. These systems lead to acyl group modifications, and to the decoration of lipid A phosphate groups with positively charged moieties with 4-amino-4-deoxy-L-arabinose (L-Ara4) or with phosphoethanolamine (pEtN). Acquired polymyxin resistance occurs chiefly through the disruption of genes involved in two-component systems [8,9]. 

In *K. pneumoniae*, alteration of the *mgrB* gene through mutations or insertion sequences removes a negative feedback on the PhoP/PhoQ regulatory system and results in polymyxin resistance. This is the mechanisms behind the epidemic dissemination of polymyxin-resistant and carbapenem resistant *K. pneumoniae*, which is associated with increased mortality, in Italian hospitals; similar findings occur among polymyxin resistant and carbapenem-resistant *K. pneumoniae* in the the United States [7,10]. In contrast, *mcr* (standing for mobile colistin resistance), a plasmid mediated genetic determinant of resistance that leads to the addition of pEtN to lipid A, appears to have had limited clinical impact [11,12]. Nevertheless, *mcr* has extended globally among *Enterobacteriaceae* of animal and human origin [13]. Resistance to polymyxins in *A. baumannii* is also due to mutations in the two-component system pmrA/pmrB leading to modification of the lipid A with pEtN [14]. Polymyxin resistance in *P. aeruginosa*, chiefly due to mutations in phoP/phoQ and pmrA/pmrB, can also result from activation of other two-component systems (parR/parS, ColR/ColS and CprR/CprS) [8]. 

There are additional mechanisms of polymyxin resistance independent of two-component systems. Importantly, the inactivation of lipid A biosynthesis genes (e.g., *lpxA, lpxC, lpxD*) has been demonstrated in certain strains of polymyxin-resistant *A. baumannii* [15]. The overexpression of outer membrane protein H (OprH), a basic protein that binds to negatively charged phosphate groups and, therefore, prevents polymyxin binding, can contribute to polymyxin resistance in *P. aeruginosa*. Similarly, polymyxin resistance can also occur through the trapping of polymyxins in the bacterial capsule of *P. aeruginosa* and *K. pneumoniae*, and through the activation of efflux pumps (e.g., AcrAB) in *K. pneumoniae* [8,9].

## 3. To Combine or Not to Combine?

The emergence of resistance adds to perennial questions surrounding polymyxins: whether their use in combination with other antibiotics results in enhanced activity against polymyxin susceptible and non-susceptible bacteria and whether this leads to improved clinical outcomes in difficult to treat infections caused by XDR Gram-negative bacteria. The rationale for the use of combination therapy against XDR Gram-negative bacteria is based on the hypothesis that polymyxins and a second antibiotic interact synergistically to increase bacterial killing and produce a combined effect greater than the sum of their separate effects, or, conversely, that the same killing effect can be achieved using lower doses of antibiotics. Results of in vitro experiments strengthen the rationale behind antibiotic combinations. Such studies demonstrate that the use of an aminoglycoside, fosfomycin, or a carbapenem, in conjunction with a polymyxin, confers additive or synergistic killing against several *P. aeruginosa* strains. Also, in vitro assays that include carbapenem-resistant *K. pneumoniae* with a broad range of polymyxin susceptibilities demonstrate the synergy of polymyxins with carbapenems, rifampicin and chloramphenicol. Similarly, in vitro studies demonstrate the synergistic killing of *A. baumannii* when a polymyxin is paired with a glycopeptide, a carbapenem, or rifampicin [16]. 

Another rationale for administering polymyxins in combination with other antibiotics is that the body of available pharmacodynamic and pharmacokinetic measurements of colistin and polymyxin B indicate that monotherapy is unlikely to reliably achieve plasma concentrations with clinical efficacy [17]. Antibiotic combinations affect diverse molecular targets and processes in bacteria, which can delay or prevent the development of resistance, which is especially relevant since monotherapy with polymyxins has resulted in regrowth of bacteria and the development of resistance during therapy [18]. The theoretical benefits of combination therapy with polymyxins need to be balanced with concerns that administering combination antimicrobial chemotherapy is likely to be more expensive and potentially more toxic than administering monotherapy. 

A recent survey asked infectious disease specialists and other clinicians from 115 hospitals in Europe and the United States to describe their management of infections caused by CRE, CRPA, and CRAB [19]. Combination therapy, usually including a polymyxin, was prescribed at least occasionally in 114 of the surveyed hospitals with the professed goals of improving effectiveness and preventing development of resistance. Unfortunately, the clinical use of polymyxins in various combinations has been studied mostly in observational retrospective cohorts of heterogeneous populations, with different bacterial genotypes and phenotypes, and compared with diverse other treatments; high-quality evidence supporting polymyxin-based combination therapy is regrettably lacking. Herein, we assess studies on the clinical efficacy of polymyxin combination therapy against infections caused by CRE, CRPA, and CRAB. 

## 4. Carbapenem-Resistant *Enterobacteriaceae*: to Combine?

Observational studies focusing on the treatment of bloodstream infections caused by CRE point to a survival advantage of combination over monotherapy (Table 1) [20,21,22,23,24]. However, even within each study, wide variations in treatment regimens exist. Monotherapy consists of either a polymyxin (polymyxin B or colistin) or tigecycline; combination regimens containing carbapenems with colistin or polymyxin B and/or tigecycline have been employed as frequently as carbapenem-sparing combinations that consisted of tigecycline and polymyxins and/or aminoglycosides. Interestingly, these studies suggested an additional benefit of including a carbapenem in combination regimens, especially when treating CRE strains with low minimum inhibitory concentration (MIC) against carbapenems. In a review of the published data on the treatment of infections caused by CRE undertaken by Tsouvelekis et al., clinical success ranged from 25% for infections caused by isolates with MIC >8 µg/mL, to approximately 70% for infections caused by isolates with MICs of 8 µg/mL or less [25,26].

A complementary dataset that permitted the evaluation of therapies against other types of serious infections caused by CRE is a large multicentric and retrospective cohort described by Tumbarello et al. [27]. In this analysis of 661 patients with various infections (447 with bloodstream infection; the remainder with lower respiratory tract, intra-abdominal structure or urinary tract infections) caused by CRE, mostly *Klebsiella pneumoniae* carbapenemase (KPC)-producing *K. pneumoniae*, combination therapy, with at least two active drugs, was associated with significantly lower 14 day mortality. Additionally, significantly higher survival rates were observed when that combination included meropenem, provided the isolate had a meropenem MIC ≤ 8 μg/mg. We note that a comparison between polymyxin monotherapy vs. polymyxins in combination with other antibiotics was unable to be performed. Other notable findings from this analysis were that septic shock, inadequate initial antibiotic therapy, and high severity of illness were associated with increased mortality in patients with CRE infections. 

Zusman et al. undertook a systematic review and meta-analysis to examine the effectiveness of polymyxin-based combination vs. monotherapy [28]. In their view, observational studies appear to show an association between polymyxin monotherapy and mortality or clinical failure in comparison with combination therapy with polymyxins and carbapenems. Moreover, an association was observed for the combination therapy of polymyxin with aminoglycosides, tigecycline, or fosfomycin and survival, especially for bacteremia with carbapenem-resistant *K. pneumoniae*. The authors warn that these associations in observational studies between polymyxin monotherapy and mortality cannot be taken as proof of the superiority of combination therapy, given the overall low quality of the evidence.

Not included in this meta-analysis was a multinational observational retrospective study that employed propensity score matching of 480 patients with CRE bloodstream infections. The INCREMENT study allowed for comparisons between the 135 patients who received combination therapy (74 of whom were treated with colistin based-regimens) and the 208 who received monotherapy (74 in this group also received colistin) [29]. Overall mortality was similar between those treated with either combination therapy or monotherapy (35% vs. 41%). This same cohort was used to derive and validate a score that measured the risk of mortality: the INCREMENT-CPE score included severe sepsis or shock at presentation, Pitt score, Charlson comorbidity index, source of bacteremia, and inappropriate empirical and early therapy [30]. Incorporating the INCREMENT-CPE score into the analysis revealed that combination therapy was associated with lower mortality (48%) than monotherapy (62%) in the high-mortality-score stratum only. 

## 5. Carbapenem-Resistant *Acinetobacter baumannii*: Not to Combine?

Regarding CRAB, the available observational evidence does not appear to suggest an advantage with combination therapy over polymyxin monotherapy [28,31,32]. Importantly, the same conclusion was derived from two open label randomized controlled trials in patients infected with CRAB who were treated with colistin in combination with rifampin vs. colistin alone, and of a preliminary trial that evaluated patients treated with colistin plus Fosfomycin, or with colistin alone [33,34,35]. The common finding in these studies, which likely underdosed colistin and included only a small number of patients, was that a difference in the primary outcome (mortality or clinical response) between combination therapy and monotherapy was not seen. Interestingly, microbiologic clearance was improved with combination therapy (Table 2) [31]. 

Paul et al. conducted a study (NCT01732250) under the auspices of the AIDA project (“assessment of clinical efficacy by a pharmacokinetic/pharmacodynamic approach to optimize effectiveness and reduce resistance for off-patent antibiotics”) in collaboration with investigators from Israel, Greece and Italy. This was a randomized controlled trial comparing colistin and meropenem against colistin alone for the treatment of serious infections caused by CRE, CRPA, and CRAB [36]. In the AIDA study approximately 90% of 406 patients enrolled had either ventilator-associated pneumonia or bloodstream infection, and 77% (*n* = 312) of infections were caused by CRAB. Although an open study, patients received standardized treatments that were pharmacologically optimized: intravenous colistin (9 million-unit loading dose, followed by 4.5 million units twice per day) or colistin with meropenem (2 g prolonged infusion three times per day). The overall result of the AIDA study was that treating with the combination of colistin and meropenem did not result in higher rates of clinical success (a composite outcome of survival, microbiological cure, hemodynamic stability, and improved oxygenation and severity of illness). Treatment with colistin monotherapy, compared with treatment with colistin and meropenem, resulted in similar mortality at 28 days (43% vs. 45%) and 14 days (32% vs. 34%); there was also not a difference between subsequent isolation of colistin-resistant bacteria (6% vs. 5%). Given that most patients in this study had CRAB, the results of the AIDA study support the conclusion that meropenem does not improve clinical outcomes when added to colistin to treat infections caused by CRAB. 

A secondary analysis of the AIDA study examined patients with infections caused by colistin resistant CRAB (MIC > 2 µg/mL determined by broth microdilution) [37]. In this subset, mortality was lower in the 52 patients infected with colistin-resistant than in the 215 with colistin-susceptible strains (42.3% vs. 52.8% at 28 days), although this difference did not reach statistical significance. The observation of decreased mortality in colistin-resistant strains suggests that colistin-resistance in CRAB may be associated with significant “fitness-cost” [38]. Furthermore, in this analysis, the combination of colistin and meropenem was associated with higher mortality among patients with colistin-resistant, but not with colistin-susceptible, CRAB, suggesting that combination therapy with colistin-meropenem may be detrimental in some instances. Therefore, it appears that patients with infections caused by colistin-resistant CRAB should be treated with other regimens (possibly in combination with colistin). In contrast with observations suggesting decreased mortality in patients infected with colistin-resistant CRAB, infection with colistin-resistant CRE (chiefly KPC-producing *K. pneumoniae*; only 1 case treated with colistin) was associated with increased risk of death [7]. These divergent observations illustrate the biological differences among different species and the pitfalls of extrapolating conclusions derived from observations in CRAB to CRE or CRPA. 

The lack of benefit of the colistin-meropenem combination against CRAB documented in the AIDA study, and the results of other smaller randomized controlled trials targeting CRAB, does not preclude further examination of other polymyxin-based combination regimens. Also, it should be noted that in the AIDA study, the number of patients enrolled with CRE (*n* = 73) or CRPA (*n* = 21) infections may have been too small to draw conclusions about the effect of colistin-meropenem combination on outcomes from infections caused by each of those pathogens. Interestingly, outcomes seemed more favorable in the subgroup of patients with infections caused by CRE treated with colistin-meropenem combination than colistin alone: 14 day mortality was 15% vs. 18%, 28 day mortality was 21% vs. 35%, and clinical failures were 46% vs. 68% [36]. These differences, however, did not reach statistical significance. Demonstrating a statistically significant difference in the composite outcome of clinical success would require enrolling approximately 150 patients with infections caused by CRE. 

## 6. Carbapenem-Resistant *Pseudomonas aeruginosa*: Less Data, More Questions

Similarly, there was a small number of patients infected with CRPA (*n* = 21) who were enrolled in the AIDA study. Therefore, the differences recorded in the AIDA study between patients with CRPA treated with colistin-meropenen and treated with colistin alone in terms of clinical failure (62% vs. 50%) and mortality (31% vs. 25%) did not reach statistical significance [36]. Further interpretation of this data is difficult given the limited number of clinical observations describing the use of polymyxins and carbapenems as combination therapy against infections caused by CRPA published in the literature [28,31]. Of note, another ongoing randomized controlled trial (NCT01597973) comparing colistin and meropenem vs. colistin alone in patients with XDR *A. baumanii*, XDR *P. aeruginosa* and CRE, may help clarify whether combination therapy with colistin and meropenem is beneficial in patients with infections caused by CRE and CRPA. It is anticipated that this study, conducted in hospitals in the United States, Israel, Bulgaria, Greece, Italy, Taiwan, and Thailand, will include approximately 444 patients by 2020. 

## 7. To Combine with Fosfomycin?

Polymyxin-based combination regimens different from colistin-meropenem may still offer benefit against CRE and CRPA. For instance, colistin was administered in combination with fosfomycin to 32 patients in the context of a “real world”, observational and prospective evaluation of fosfomcyin for the treatment of serious infections caused by CRE (*n* = 41) and/or CRPA (*n* = 17), 54.2% of whom had a successful clinical outcome [39]. Michalopoulos et al. also reported favorable clinical and microbiological outcomes in 11 patients with carbapenemase-producing *K. pneumoniae* treated with intravenous fosfomycin, six of whom received fosfomycin combined with colistin [40]. Apisarnthanarak and Mundy conducted a retrospective study comparing colistin and fosfomycin (*n* = 24) vs. colistin and doripenem (*n* = 25) for the treatment of patients with hospital-acquired and ventilator associated pneumonia caused by CRPA; mortality in both groups was 40% [41]. These studies have clear limitations stemming from their observational nature and the small number of subjects included, but they can serve to inform the design of future studies that may provide evidence of higher quality. 

## 8. Is it Still a Relevant Question?

The question of how to optimize polymyxin-based regimens, including the exploration of combination regimens, remains relevant despite the development of new antibiotics against XDR Gram-negative bacteria. Ceftazidime-avibactam, imipenem-relebactam and meropenem-vaborbactam offer activity against CRE that produce serine carbapenemases (e.g., KPC-2, KPC-3, and OXA-48), while ceftolozane-tazobactam stands as an option to treat CRPA in the absence of acquired carbapenemases. Other antibiotics may be of future value as well, such as cefiderocol, plazomycin, and eravacycline. Clinical experience supporting the use of ceftazidime-avibactam and ceftolozane-tazobactam instead of colistin-based regimens for the treatment of CRE and CRPA, respectively, is accumulating [42,43]; a definite benefit of using these agents is reducing the risk of kidney injury, which affects at least a third of patients treated with colistin [44]. However, these new antibiotics do not address the treatment of infections caused by strains of CRE and CRPA in which carbapenem resistance is mediated by metallo-beta-lactamases (e.g., New Delhi metallo-beta-lactamase (NDM), Verona integron-mediated metallo-beta-lactamase (VIM)), and neither do they offer activity against CRAB. Additionally, new agents are not universally available, and the predictable emergence of resistance has already occurred [45,46,47,48]. 

Polymyxin-based “salvage regimens” may remain necessary for patients with serious infections caused by XDR Gram-negative bacteria, including polymyxin resistant strains. Unfortunately, when clinicians encounter cases that pose therapeutic challenges, they cannot find solace, nor solutions for their patients, under the rubric of “insufficient evidence”. Rather, clinicians must integrate their understanding of the mechanisms of resistance and of the activity of novel combinations to respond to difficult clinical scenarios, and stand to learn by carefully evaluating individualized treatments. Case reports that describe these approaches, including, for instance, the combination of ceftazidime-avibactam and aztreonam to treat infections caused by metallo-beta-lactamase harboring bacteria, are very valuable. On the one hand, case reports serve to probe the validity of the mechanistic rationale underlying novel therapeutic approaches; on the other hand, case reports also help clinicians identify valid options for their patients. It is clear, however, that case reports do not replace rigorous attempts at collating scientific and clinical evidence [49,50,51,52]. 

Studies in vitro that assess various combinations of antibiotics using time-kill methods and hollow fiber infection models can also offer a refined “proof of concept” for the use of polymyxin-based combination therapies that might be considered in the clinical setting, as has been demonstrated for the combination of polymyxin and fosfomycin against KPC-producing *K. pneumoniae* [53]. In another example, the simulation of human pharmacokinetics using the hollow fiber infection model demonstrated the activity of an optimized combination involving polymyxin B, aztreonam, and amikacin that eradicated a formidable strain of CRE harboring a metallo-beta-lactamase, NDM-5-producing *Escherichia coli*, that also harbored the plasmid-mediated mechanism of polymyxin resistance, *mcr-1* [54]. 

## 9. How Can We Make Polymyxin-based Combinations Work?

The current paradigm dictates that pharmacologic modeling of antibiotics anticipates the results of experiments in humans and predicts what will occur in the clinic. Thus, investigators can be confident that doses of antibiotics administered to patients with known characteristics will effectively treat infections at a given site caused by bacteria with certain MICs. Nevertheless, at least in the case of polymyxin-based combinations, there appears to be a gap between the predictions derived from in vitro models and the results of clinical trials, which to date have not clearly demonstrated the benefit of combination therapy. 

We venture that this is due to the unpredictable pharmacokinetics of colistin, with substantial interpatient variations in the average steady-state plasma concentration occurring especially among critically ill patients [55], and to the intractable mixture of comorbidities and severe acute illness encountered in patients infected with XDR Gram-negative bacteria. Furthermore, these patients are often infected in the respiratory tract, a compartment where polymyxins (and other classes of antibiotics) do not reliably penetrate [56,57]. Additionally, XDR Gram-negative bacteria are often not suspected at the onset of illness and patients can experience significant delays in the administration of effective therapy; for instance, in the AIDA study, only half the patients received appropriate empirical antibiotic treatment within 2 days [36]. Although the impact of effective antibiotic treatment is highly variable, a systematic review and meta-analysis estimated a pooled adjusted odds ratio of 1.6 for all-cause mortality of appropriate empirical antibiotic treatment during the first 2 days [58]. 

Perhaps combinations regimens that include carefully dosed polymyxin B, which appears to have less variations in the average steady-state plasma concentration, will fare better than colistin for the treatment of bloodstream infections [55]. However, since colistin is mainly eliminated by the kidneys and high levels of colistin are found in the urinary tract, colistin may be a better option than polymyxin B for the treatment of infections of the urinary tract [4]. 

It is also foreseeable that the implementation of rapid and precise diagnosis of XDR pathogens using molecular methods, followed by correct determination of polymyxin MICs, will facilitate the prompt administration of effective therapies. The measurement of antibiotic levels in critically ill patients and other vulnerable patient populations is also necessary. Therapeutic drug monitoring (TDM) of polymyxins can “close the loop” and provide real-time pharmacokinetic and pharmacodynamic parameters in patients with XDR Gram-negative bacterial infections treated with polymyxins [4,59]. Indeed, adoption of these tools is the basis for personalized antibiotic chemotherapy, or “precision medicine”, which may require a radical transformation of how we administer antibiotics to treat serious infections caused by XDR Gram-negative bacteria [20]. 

Even then, the use of polymyxin-based combinations will require validation in randomized trials adapted to the challenges of studying patients with XDR Gram-negative bacteria, but that still ensure the comparison of standardized treatment regimens and minimize confounding by indication and survival treatment selection bias [60]. Such efforts are essential to achieve optimal treatment strategies for infections caused by XDR Gram-negative bacteria. 

## 10. Conclusions

Despite the paucity of high-quality evidence supporting the superiority of polymyxin-based combination therapy vs. polymyxin monotherapy, clinicians who rely on polymyxins to treat serious infections caused by XDR Gram-negative bacteria often employ them in combination with other antibiotics. However, some certainties are emerging: a recent randomized controlled trial demonstrated that the combination of colistin and meropenem is not superior to colistin monotherapy for the treatment of infections caused by CRAB, while results are not conclusive for CRE and CRPA. The observational studies that suggest a benefit of colistin and carbapenem combination therapy against infections caused by CRE, especially KPC-producing *K. pneumoniae* with low carbapenem MICs in severely ill patients, are limited due to confounding by indication and allocation bias (i.e., differences between characteristics of the groups influence both treatment and outcomes), and due to the non-standardized administration of heterogeneous regimens. Limited clinical data and in vitro pharmacologic modeling suggest that polymyxins-based combinations may yet prove valuable against CRE and CRPA as part of “salvage regimens” against XDR Gram-negative bacteria in cases where there is a lack of other therapeutic options. Demonstrating their potential benefit will require clinical trials and practices that incorporate careful attention to polymyxin dosing, rapid detection of resistance and MIC determinations, and therapeutic drug monitoring.

## Figures and Tables

**Table 1:** Observational studies with data on polymyxin combination therapy and monotherapy for bloodstream infections caused by carbapenem-resistant *Enterobacteriaceae* [15].

Reference	Country/CRE	Combination Therapy			Monotherapy		
	Genotype	Regimens	No. of Patients	Survival Rate (%)	Antibiotic	No. of Patients	Survival Rate (%)
Daikos et al., 2014 [23]	Greece/KPC and VIM-producing *K. pneumoniae*	Carb-Tig-AG or Col	11	100	Col	22	45.5
		Carb-Col	7	57.2			
		Tig-AG-Col	11	73			
		Tig-Col	21	76.2			
		AG-Col	17	70.6			
		Other	36	67	Other	50	60
Qureshi et al., 2012 [24]	United States/KPC-producing *K. pneumoniae*	Col-Carb	5	80	Col	7	43
		Col-Tig	1	100			
		Col-FQ	1	100			
		Other	8	88	Other	12	42
Tumbarello et al., 2012 [22]	Italy/KPC-producing *K. pneumoniae*	Tig-Col	23	70	Col	22	50
		Col-AG	7	43			
		Tig-Col-Carb	16	87			
		Col-AG-Carb	1	0			
		Other	32	59.4	Other	24	41.7
Zarkotou et al., 2011 [21]	Greece/KPC-producing *K. pneumoniae*	Tig-Col	9	100	Col	7	43
		Tig-Col-Carb	2	100			
		Tig-Col-AG	1	100			
		Col-AG	2	100			
		Other	5	100	Other	8	62.5

CRE, carbapenem-resistant *Enterobacteriacea;* KPC, *Klebsiella pneumoniae* carbapenemase; VIM, Verona integron-mediated metallo-beta-lactamase; Col, colistin; Tig, tigecycline; Carb, carbapenem; AG, aminoglycoside; FQ, fluoroquinolone.

**Table 2:** Open label randomized controlled trials comparing polymyxin combination antibiotic therapy vs. monotherapy for the treatment of infections caused by carbapenem-resistant *Acinetobacter baumannii* [16].

Reference	Country, Dates, Number and Types of Patients	Monotherapy	Combination Therapy	Primary Outcome	Results
Durante-Mangoni et al., 2013 [34]	Italy, November 2008–July 2011 - *n* = 209 - BSI, HAP, VAP, IAI - Duration: 10–21 days	Colistin 2 million units IV every 8 h	Rifampicin 600 mg every 12 h + Colistin	30 day all-cause mortality	- 45/104 (43.3%) mortality with combination vs. 45/105 (42.9%) with monotherapy (NS) - Microbiologic eradication: 63/104 (60.6%) with combination vs. 47/105 (44.8%) with monotherapy (*p* = 0.034)
Sirijatuphat and Thamlikitkul, 2014 [35]	Thailand, January 2010–March 2011 - *n* = 94 - Pneumonia, BSI, UTI, SSTI, IAI - Duration: 7–14 days	Colistin base activity 5 mg/kg/ day	Fosfomycin 4 g IV every 12 h + Colistin	Favorable clinical outcome: “cure or improvement at 28 days”	- 62.8% favorable clinical outcome with combination vs. 56.4% with monotherapy (NS) - Microbiologic eradication: 100% with combination vs. 84.5% with monotherapy (*p* = 0.023)
Aydemir et al., 2013 [33]	Turkey, March 2011–March 2012 - *n* = 43 - VAP	Colistin base activity 300 mg daily	Rifampicin 600 mg daily + Colistin	Clinical response: no fever, normal respiratory secretions, PaO2/FiO2>240 or no mechanical ventilation	- 11 (52.4%) clinical response with combination vs. 9 (40.9%) with monotherapy (NS) - Time to microbiologic clearance: 3.1 +/−0.5 days with combination vs. 4.5+/−1.7 days with monotherapy (*p* = 0.029)
Paul et al., 2018 [36]	March 2013–February 2017 Greece, Israel, Italy - *n* = 312 (patients with CRAB) - BSI, HAP, VAP, UTI - Duration: 10 days	Colistin 9 million units loading IV once then 4.5 million units every 12 h	Meropenem 2 g IV prolonged infusion every 8 h + Colistin	Clinical success at 14 days: composite of survival, hemodynamic stability, improved/stable SOFA, improved/stable PaO2/FiO2 (HAP/VAP), negative blood cultures (BSI).	- 19% clinical success with combination vs. 17% with monotherapy (NS) - 46% mortality at 28 days with combination vs. 52% with monotherapy

BSI Bloodstream Infection; HAP Hospital-Acquired Pneumonia; VAP Ventilator-Associated Pneumonia; UTI Urinary Tract Infection; IAI Intrabdominal Infection; SSTI Skin and Soft Tissue Infection; SOFA Sequential Organ Failure Assessment score; NS non-significant difference (*p* > 0.05).
